# Evaluation of the synergistic effects of curcumin-resveratrol co-loaded biogenic silica on colorectal cancer cells

**DOI:** 10.3389/fphar.2024.1341773

**Published:** 2024-06-11

**Authors:** Adriana Ochoa-Sanchez, Padmavati Sahare, Surajit Pathak, Antara Banerjee, Miriam Estevez, Asim K. Duttaroy, Gabriel Luna-Bárcenas, Sujay Paul

**Affiliations:** ^1^ NatProLab, School of Engineering and Sciences, Tecnologico de Monterrey, Queretaro, Mexico; ^2^ Institute of Advanced Materials for Sustainable Manufacturing, School of Engineering and Sciences, Tecnologico de Monterrey, Queretaro, Mexico; ^3^ Chettinad Academy of Research and Education (CARE), Chettinad Hospital and Research Institute (CHRI), Department of Medical Biotechnology, Faculty of Allied Health Sciences, Chennai, India; ^4^ Centre of Applied Physics and Advanced Technologies (CFATA), National Autonomous University of Mexico, Queretaro, Mexico; ^5^ Department of Nutrition, Institute of Basic Medical Sciences, Faculty of Medicine, University of Oslo, Oslo, Norway

**Keywords:** colorectal cancer, curcumin, resveratrol, bioformulation, biogenic silica, therapy

## Abstract

Colorectal cancer (CRC) remains a significant global health concern, being the third most diagnosed cancer in men and the second most diagnosed cancer in women, with alarming mortality rates. Natural phytochemicals have gained prominence among various therapeutic avenues explored due to their diverse biological properties. Curcumin, extracted from turmeric, and resveratrol, a polyphenol found in several plants, have exhibited remarkable anticancer activities. However, their limited solubility and bioavailability hinder their therapeutic efficacy. To enhance the bioavailability of these compounds, nanomaterials work as effective carriers with biogenic silica (BS) attracting major attention owing to their exceptional biocompatibility and high specific surface area. In this study, we developed Curcumin-resveratrol-loaded BS (Cur-Res-BS) and investigated their effects on colorectal cancer cell lines (HCT-116 and Caco-2). Our results demonstrated significant concentration-dependent inhibition of cell viability in HCT-116 cells and revealed a complex interplay of crucial proto-onco or tumor suppressor genes, such as *TP53*, *Bax*, *Wnt-1*, and *CTNNB1*, which are commonly dysregulated in colorectal cancer. Notably, Cur-Res-BS exhibited a synergistic impact on key signaling pathways related to colorectal carcinogenesis. While these findings are promising, further investigations are essential to comprehensively understand the mechanisms and optimize the therapeutic strategy. Moreover, rigorous safety assessments and *in vitro* studies mimicking the *in vivo* environment are imperative before advancing to *in vivo* experiments, ensuring the potential of Cur-Res-BS as an efficient treatment for CRC.

## 1 Introduction

Colorectal carcinoma (CRC), encompassing colon and rectum cancer, holds the alarming status of being the third most frequently diagnosed cancer (IARC, 2020). Even more concerning is its position as the second-leading cause of cancer-related deaths worldwide, responsible for nearly 2 million new cases and 1 million deaths in 2020, with a rising incidence observed in developing nations (Morgan et al., 2023). CRC typically originates in the glandular epithelial cells of the large intestine and represents a complex disorder influenced by genetic and epigenetic mutations across various signaling pathways (Akhtar et al., 2020). Extensive research efforts have been dedicated to unraveling the intricate mechanisms underlying CRC (Emam et al., 2022; Abd El Fattah et al., 2023). Signaling pathways such as Bcl-2, Wnt/β-catenin, p53, ERK/MAPK, and NF-κB pathways have been pivotal subjects of investigation. These pathways play crucial roles in processes like proliferation, invasion, progression and apoptosis inhibition in CRC cells (Koveitypour et al., 2019). Specific genes within these pathways, such as *Bax*, *Bcl-2*, *APC*, *Wnt-1*, *TP53*, *CTNNB1*, and *EGFR*, among others, have been identified for their significant involvement in CRC progression (Pricci et al., 2020). Mutations in these genes lead to the development of hyperactive cells characterized by increased replication and survival, ultimately resulting in the formation of benign adenomas. Over time, these adenomas can progress into carcinomas and eventually metastasize over several decades (Rawla et al., 2019).

While advancements in early detection and treatment have contributed to reduced mortality rates, the emergence of drug-resistant cancers and adverse side effects associated with current treatments emphasize the pressing need for innovative and highly efficient therapeutic agents (Giordano and Tommonaro, 2019).

Phytochemicals, including flavonoids, alkaloids, phenolics, glycosides, carotenoids, glucosinolates, anthraquinones, and nitrogenous derivatives, are present in various plant parts (Akhtar et al., 2020). These compounds have demonstrated remarkable biological properties, such as antimicrobial, anti-inflammatory, analgesic, antioxidant, antitumor, and anticancer activities, making them valuable candidates for the treatment and prevention of several chronic diseases (Garcia-Oliveira et al., 2021). Phytochemicals manifest their anticarcinogenic effects through diverse mechanisms (Das et al., 2023). Curcumin, a polyphenolic compound extracted from turmeric (*Curcuma longa*), has garnered considerable scientific interest since its initial extraction in 1870 (Pricci et al., 2020). Over recent decades, extensive research has explored its unique properties, encompassing antioxidant, anti-inflammatory, antiandrogenic, anti-tumor, antiproliferative, antimicrobial, hypoglycemic, and chemopreventive activities, leading to its recognition as a multifaceted therapeutic agent (Tomeh et al., 2019). Notably, its remarkably minimal toxicity allows for safe consumption, with no reported side effects, even at doses as high as 10 g per day (Mansouri et al., 2020). In the realm of anticarcinogenic effects, curcumin works through diverse mechanisms, including the regulation of several cellular pathways (Kabir et al., 2021). Numerous studies conducted over the years have evidenced that the modulation of these signaling pathways can suppress the activity of proteins and cytokines associated with cancer initiation, progression, proliferation, and tumor development. Additionally, curcumin can induce the activity of other proteins implicated in apoptosis or autophagy (Mansouri et al., 2020; Ojo et al., 2022). Curcumin has been found to downregulate the Wnt/β-catenin pathway, which is overactive in cancer and leads to abnormal cell proliferation and tumor growth. This effect could be achieved by modulating specific genes like *Wnt-1*, *APC*, *CCND1*, *EGFR*, and *CTNNB1* (Vallée et al., 2019). Additionally, the p53 pathway is commonly disrupted in cancer due to *TP53* gene dysregulation. Studies have shown that curcumin can regulate the expression levels of p53 pathways (Ismail et al., 2019).

Resveratrol (trans-3,4,5-trihydroxystilbene), a naturally occurring polyphenol belonging to the stilbene class, is synthesized by approximately 72 different plant species, including those within the spermatophytes family such as peanuts, grapes, mulberries, cranberries, blackberries, and blueberries (Gianchecchi and Fierabracci, 2020; Talib et al., 2020). Plants produce resveratrol as a natural defense mechanism in response to external stimuli, including biotic infections, physical injury, ultraviolet radiation, and excessive sunlight exposure (Vervandier-Fasseur and Latruffe, 2019). Initially identified in 1939 from the white hellebore plant (*Veratrum grandiflorum*), resveratrol has since garnered significant attention due to its diverse therapeutic properties, which include antioxidative, anti-proliferative, antimicrobial, anti-inflammatory, and anti-neurodegenerative effects (Chen et al., 2022). Resveratrol modulates multiple signaling pathways associated with cell growth, division, angiogenesis, apoptosis, metastasis, autophagy, and chemotherapy resistance (Tian et al., 2019). Like curcumin, resveratrol can also influence the Wnt/β-catenin signaling pathway, a crucial network that regulates cell cycle, proliferation, migration, and differentiation. Treatment with resveratrol has been demonstrated to reduce the expression of Wnt-1 and CTNNB1, genes linked to proliferation (Pashirzad et al., 2021). Moreover, resveratrol exhibited a potent pro-apoptotic impact in CRC, as evidenced by an elevated Bax/Bcl-2 ratio, heightened p53 levels, and activation of caspase-3 and -8 (Gavrilas et al., 2019). Recent studies emphasize a synergistic interaction upon combining curcumin and resveratrol, resulting in decreased survival of cancer cells (Arena et al., 2021). The synergy between curcumin and resveratrol emanates from their cytotoxic properties, inducing apoptosis and autophagy through diverse signaling pathways (Zheng et al., 2022). Even with the mentioned advantages, the therapeutic efficacy of curcumin and resveratrol is impeded by multiple limitations (Tomeh et al., 2019). Principal among these challenges are their poor solubility, hydrophobic characteristics, weakened cellular uptake, and significant first-pass effect. These factors collectively reduce bioavailability and chemical stability (Kabir et al., 2021).

Nanotechnology, a rapidly evolving research field, focuses on manipulating functional systems at a nanoscale (Wilczewska et al., 2012; Sahare et al., 2022). Bioformulation-based delivery systems have found extensive applications, including gene therapy, antibiotic delivery, vaccine administration, and protein delivery, illustrating their multifaceted utility (Kumar et al., 2021). Bioformulation-based delivery systems offer several advantages, including non-toxicity and biodegradability, and their small size enables diverse administration routes such as intravenous, nasal, oral, and intraocular, among others (Kumar et al., 2021). Furthermore, these systems enhance drug biodistribution and efficacy by specifically targeting cancer cells, thus minimizing undesirable side effects associated with off-target deliveries (Bamrungsap et al., 2012). Bioformulations also safeguard the therapeutic properties of drugs, preventing rapid elimination or degradation within the body. This preservation results in higher drug concentrations in target tissues, maximizing the therapeutic impact (Wilczewska et al., 2012).

For delivery systems, bioformulations must be non-toxic and biocompatible. In this context, BS have gained attention as promising nanocarriers due to their unique attributions, including high loading capacity, extensive surface area, and large pore size ranging from 2 to 50 nm (Lu et al., 2007; Bharti et al., 2015). *Equisetum miryochaetum*, a horsetail species native to South America, is crucial in this study because of its unique silica accumulation pattern. In this plant, silica is concentrated in dense thickenings, forming clusters dispersed across the plant’s surface. Cytotoxicity assessments have confirmed the biocompatibility of BS with the human body, demonstrating their biodegradability and low toxicity. Significant positive outcomes have also been observed in biodistribution and excretion evaluations.

The aim of this research, which constitutes the initial phase of a broader study, is to examine the synergistic therapeutic effects of Curcumin-Resveratrol co-loaded BS on Colorectal Cancer Cell lines. This work represents the first endeavor to investigate the therapeutic benefits of curcumin-resveratrol-loaded plant-based BS on CRC cells. This investigation also involves assessing the expression levels of proto-onco or tumor suppressor genes such as *TP53*, *Bax*, *Wnt-1*, and *CTNNB1*. Understanding the molecular alterations induced by the bioformulation in these key genes is crucial for establishing the groundwork for subsequent phases of the study.

## 2 Materials and methods

### 2.1 Materials

Curcumin, resveratrol, 3-aminopropyltriethoxysilane (APTES), Glutaraldehyde (GTA), Dulbecco’s Modified Eagle Medium (DMEM), Fetal Bovine Serum (FBS), and 3-(4,5-dimethylthiazol2-yl)-2,5-diphenyltetrazolium bromide (MTT) were purchased from Sigma-Aldrich (Massachusetts, United States). Human colorectal carcinoma cell lines HCT-116 and Caco-2 and Human Embryonic Kidney (HEK) 293 cells were obtained from the American Type Culture Collection (ATCC). Oligos (*Bax*, *CTNNB1*, *Wnt-1*, *TP53*, and *GAPDH*) were provided by Merck (Massachusetts, United States).

### 2.2 Extraction of BS

In this study, the *Equisetum myriochaetum* plant was used for the extraction of BS following a previously described protocol (Sola-Rabada et al., 2018) with slight modifications. The plant material, comprising stems and branches, was separated and sundried for 72 h and subsequently ground into a fine powder. The extraction of BS was performed through an acid digestion method using concentrated HNO_3_/H_2_SO_4_ (4:1) solution. Specifically, 20 g of dry plant material was mixed with 1.875 L of the acid solution and stirred on a hot plate at 70°C for 48 h until a fine white precipitate was formed. This precipitate was separated, washed multiple times with copious amounts of distilled water until reaching a neutral pH, and then subjected to lyophilization that yielded 16.65% of BS as white powder.

### 2.3 Curcumin and resveratrol immobilization

Three mg of curcumin were immobilized onto 200 mg of BS through a silanization process using 3-aminopropyltriethoxysilane (APTES). Briefly, BS was first incubated in 5% APTES in ethanol solution for 2 h, followed by annealing at 110°C for 15 min. Subsequently, curcumin was dissolved in ethanol, added to the BS, and kept in a shaker for 2 h at 90 rpm, followed by ethanol wash. To load resveratrol into the BS, the obtained curcumin-loaded BS sample was treated with 25% Glutaraldehyde (GTA) in ethanol, stirred for 1 h at 90 rpm and washed with ethanol. 3 mg of resveratrol was then added to the sample and stirred for 3 h at 90 rpm. All the immobilization was performed in cold and dark conditions.

### 2.4 Characterization of BS (pre and post immobilization)

The surface morphology of the BS was analyzed using scanning electron microscopy (SEM). Samples were prepared by placing them onto a copper holder with a Colloidal Graphite alcohol base (Electron Microscopy Sciences, Pennsylvania, United States) and subsequently dried at 50°C. SEM images were captured for detailed observation using Hitachi High-Tech’s scanning electron microscope SU3800/SU3900 (Tokyo, Japan). The X-ray diffraction (XRD) technique was employed to evaluate the crystallinity of the samples, utilizing Cu Kα radiation with a wavelength of 1.54056 Å in a PANalytical X’Pert PRO instrument (Worcestershire, United Kingdom). Ground samples were densely packed into aluminum sample holders and scanned from 5° to 80° at 2θ at room temperature. The procedure involved an accelerating voltage of 45 kV, a filament current of 40 mA, and a scan speed of 0.02° per second. The organic content analysis was conducted using Thermogravimetric Analysis (TGA) with a TGA/DSC 2 STARe (Mettler Toledo, Greifensee, Switzerland). 5.5 mg of the sample was loaded into an aluminum holder and subjected to heating with a ramp rate of 10°C/min, ranging from 25°C to 900°C, under a nitrogen atmosphere with a flow rate of 40 mL/min. An Anton Paar Litesizer 500 DLS analyzer was used for the particle size and zeta potential measurements of the extracted BS. The temperature was set at 25°C and the equilibrium time was 2 minutes. For particle size, the optimum angle was automatically obtained for each sample based on transmittance. At the same time, the zeta potential was taken and calculated by the Smoluchowski approximation. The physical properties of BS, such as specific surface area, pore volume, and pore size were determined using the gas adsorption method. Brunauer-Emmett-Teller (BET) was used for the calculation of specific surface area, while the Barrett-Joyner-Halenda (BJH) method was employed to obtain the pore size distribution, where a five-point adsorption isotherm was applied within the relative pressure range of 0.05–0.3 at 77.35 K (Bawa et al., 2011). For the identification and characterization of functional groups onto the BS, IR spectra of samples were recorded using a Fourier transform infrared spectrometer (FT-IR Spectrometer PerkinElmer, Massachusetts, United States). The scanning range used was 650–4,000 cm^−1^ with 12 scans, and the resolution was set at 4 cm^−1^.

### 2.5 Determination of encapsulation efficiency and drug release by UV-Vis spectroscopy

The actual loading capacity of each phytochemical (curcumin and resveratrol) was calculated by utilizing a Genesys 10S UV-VIS spectrophotometer (Thermo Fisher Scientific, Massachusetts, United States). The encapsulation efficiency (EE) of three bioformulations was determined: Cur-BS, Res-BS, and Cur-Res-BS. Prior to use, calibration curves were established for the two phytochemicals, curcumin and resveratrol, both dissolved in ethanol. The curcumin calibration curve was plotted at a concentration range from 100 to 1,000 μg/mL, whereas the resveratrol curve spanned from 150 to 1,500 μg/mL. To determine the amounts of curcumin and resveratrol loaded within the pores of the BS, the optical density of the supernatant obtained during the bioformulations preparation was measured at 425 nm (for curcumin) and 303 nm (for resveratrol), while for the Cur-Res-BS bioformulation, the amount of curcumin and resveratrol were measured simultaneously using a spectrophotometer with their respective wavelengths. The concentration of unbound components in the supernatant and washes was then calculated using the previously established calibration curve. EE (%) was calculated as follows:

$$DEE=\left[\frac{Total\;Cur\;and\;Res\;amount-Cur\;and\;Res\;in\;supernatant}{Total\;Cur\;and\;Res\;amount}\right]x\;100$$

*In vitro* drug release assay was adapted from the previously reported protocol (Saputra et al., 2022) with slight modification. 5 mg of phytochemicals-loaded bioformulations was suspended into 50 mL beakers containing 10 mL of phosphate buffer saline (1M) solution maintained at 37°C and uniformly stirred. To observe the effect of pH on the release of phytochemicals, the release assay was conducted at two different pH values: 7.4 and 5.6. For the quantification of the released phytochemicals, 1 mL of sample aliquots were withdrawn at the predetermined time intervals, centrifuged at 10,000 rpm for 5 min and the supernatant was used for measuring the absorbance of the solutions using UV-Visible spectroscopy at the wavelengths of 425 nm for curcumin and 303 nm for resveratrol, respectively (Nasr and Abdel Rahman, 2019). The pellet was washed with buffer after the measurement, redispersed in 1 mL buffer, and returned back to the assay system to maintain the sink condition. The assay was performed in triplicate and the calculations were done using the calibration curves.

### 2.6 Cell culture

In this study, a human colorectal carcinoma cell line (HCT-116) and a human colon carcinoma cell line (Caco-2) were employed. The cells were cultured in Dulbecco’s Modified Eagle Medium (DMEM) with high glucose, supplemented with 10% Fetal Bovine Serum (FBS). To maintain the desired cell confluence, all cultures were kept in a 5% CO_2_ humidified incubator at 37°C.

### 2.7 Cell viability assay

The viability of the cells in the presence of the free phytochemicals and immobilized bioformulation was analyzed using 3-(4,5-dimethylthiazol2-yl)-2,5-diphenyltetrazolium bromide (MTT) assay (Sigma-Aldrich, Massachusetts, United States) HCT-116 and Caco-2 cells were cultured in 24-well plates at a seeding density of 10,000 cells per well for 24 h at 37°C and 5% CO_2_ to get confluency of 80%. Cells were treated with three treatments: Cur-BS, Res-BS, and Cur-Res-BS. Six different concentrations of the treatments ranging from 100 to 1,000 μg/mL were added to the cells. For controls, the cells remained without bioformulation (or without any phytochemicals), and only the medium was replaced. After the plates were incubated for 24 h, 300 μL of MTT (5 mg/mL) were added to each well, and after 4 h of incubation, the crystals were dissolved with 80% ethanol and triplicates were transferred to a 96-well plate. The absorbance was measured with a microplate reader xMark Bio-Rad Laboratories (California, United States) at 570 nm, and the IC_50_ dose was determined. The experiment was repeated until the IC_50_ dose calculation followed a 48 h incubation period with the treatment. All the study was performed in triplicates.

Cell viability was calculated by the following formula:

$$Cell\;viability=\left[\frac{{OD}_{treated\;cells}}{{OD}_{control\;cells}}\right]x\;100$$

Cell viability experiments were carried out on HCT-116 and Caco-2 cell lines to evaluate the toxic effects of free curcumin and resveratrol at concentrations ranging from 100 to 1,000 μg/mL. The protocols used in these experiments precisely mirrored those applied in assessing the cytotoxicity of the bioformulations. Additionally, the biocompatibility of the extracted BS with the cells was confirmed by examining the cytotoxicity of BS at concentrations of 500 and 1,000 μg/mL on both cell lines. The parameters for this analysis remained consistent with those utilized for the bioformulations. Furthermore, cell viability assessment of Cur-Res-BS bioformulation at concentrations ranging from 100 to 1,000 μg/mL was conducted on Human Embryonic Kidney (HEK) 293 cells to gauge the impact of the bioformulation on healthy cells. The procedures for this experiment were identical to those employed in evaluating the cytotoxicity of the bioformulations on colorectal cancer cell lines. Following this, the Selectivity Index (SI) was derived by calculating the ratio of IC50 values between HEK-293 healthy cells and HCT-116 cancer cells using the following formula:

$$SI=\frac{{IC}_{50\;}of\;the\;bioformulation\;on\;HEK293}{\;{IC}_{50\;}of\;the\;bioformulation\;on\;HCT116}$$

### 2.8 RNA extraction and cDNA synthesis

Gene expression in HCT-116 was assessed using a previously determined IC50 dose value. HCT-116 cells were seeded at a density of 1 × 10^4^ (cells/well) in 24-well plates and exposed to a combined treatment (Cur-Res-BS) at a concentration of 381 μg/mL and incubated for 24 and 48 h. Untreated cells served as controls. RNA extraction and purification from both treated and untreated HCT-116 cells were performed using the miRNeasy Tissue/Cells Advanced Micro Kit (Qiagen, Hilden, Germany) following the manufacturer’s protocol. The concentration and purity of the extracted RNA were determined using a NanoDrop One spectrophotometer (Thermo Fisher Scientific, Massachusetts, United States). First-strand cDNA was synthesized from the total RNA using the First-Strand cDNA Synthesis kit (Takara Bio, United States) as per the manufacturer’s instructions. The resulting cDNA was diluted to a final volume of 100 µL in RNase-free water and stored at −80°C for subsequent use.

### 2.9 Expression analysis of target genes by RT-qPCR

The expression of four target genes (*TP53*, *Wnt-1*, *CTNNB1* (*β-catenin*), and *Bax*), recognized for their pivotal roles in carcinogenesis based on previous research, was evaluated using the StepOne Real-Time PCR System (Applied Biosystems, California, United States) and the TB Green Advantage qPCR premixes Kit (Tokyo, Japan) as per the manufacturer’s guidelines. Primers were purchased from Merck (Massachusetts, United States). To enhance the statistical robustness of the results, three biological replicates and three technical replicates were analyzed during the qPCR analysis. The resulting cycle threshold (Ct) values were processed using the 2^(−ΔΔCt)^ method, using *GAPDH* as the reference control.

### 2.10 Statistical analysis

The statistical significance of the differences between the two groups derived from the three biological replicates of each experiment at two different time points was evaluated using Two-Way ANOVA. To evaluate statistical significance, a *p*-value of less than 0.05 was employed (*p < 0.05). The findings were expressed as the mean value ± standard deviation (Mean ± SD).

## 3 Results

### 3.1 Synthesis and preparation of Cur-Res-BS

**FIGURE 1:**
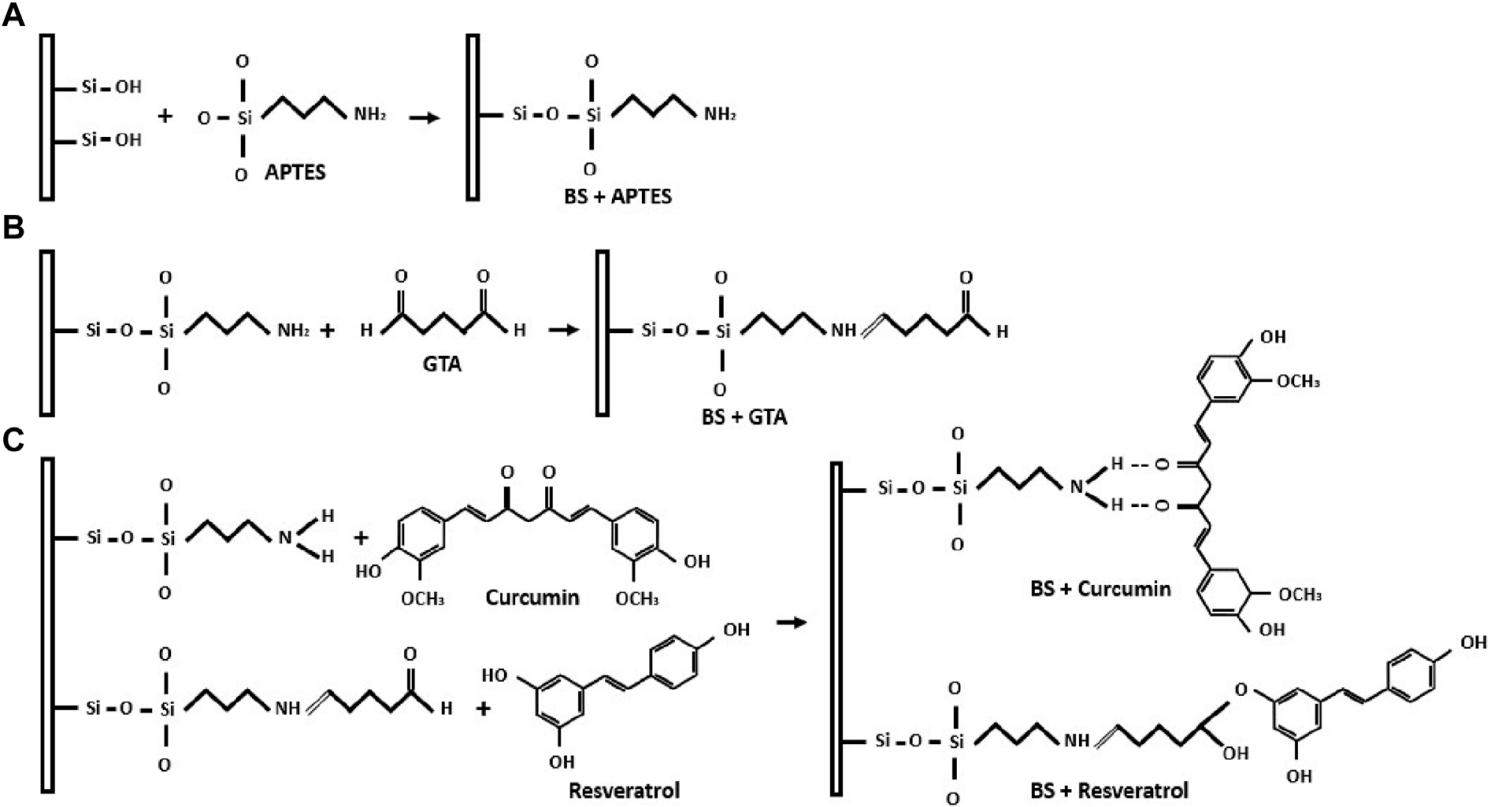
Schematic representation demonstrates the BS functionalization and binding process. **(A)** Surface modification of BS using APTES through silanization. **(B)** Addition of aldehyde groups to silanized BS using GTA. **(C)** Formation of covalent bonds between curcumin, resveratrol, and the functionalized BS.

BS were synthesized using the acid digestion method derived from the *Equisetum myriochaetum* plant (common name: Mexican giant horsetail). As illustrated in Figure 1, the BS was functionalized using APTES, a silane coupling agent, to introduce an amine-terminated group. Subsequently, GTA was immobilized to the silanized samples, adding an aldehyde group. Covalent bonds facilitated the immobilization of Curcumin and Resveratrol onto the functionalized samples. This process led to the development of three distinct bioformulations: Cur-BS, Res-BS, and Cur-Res-BS.

### 3.2 Fourier transform infrared spectrometer (FTIR) analysis

**FIGURE 2:**
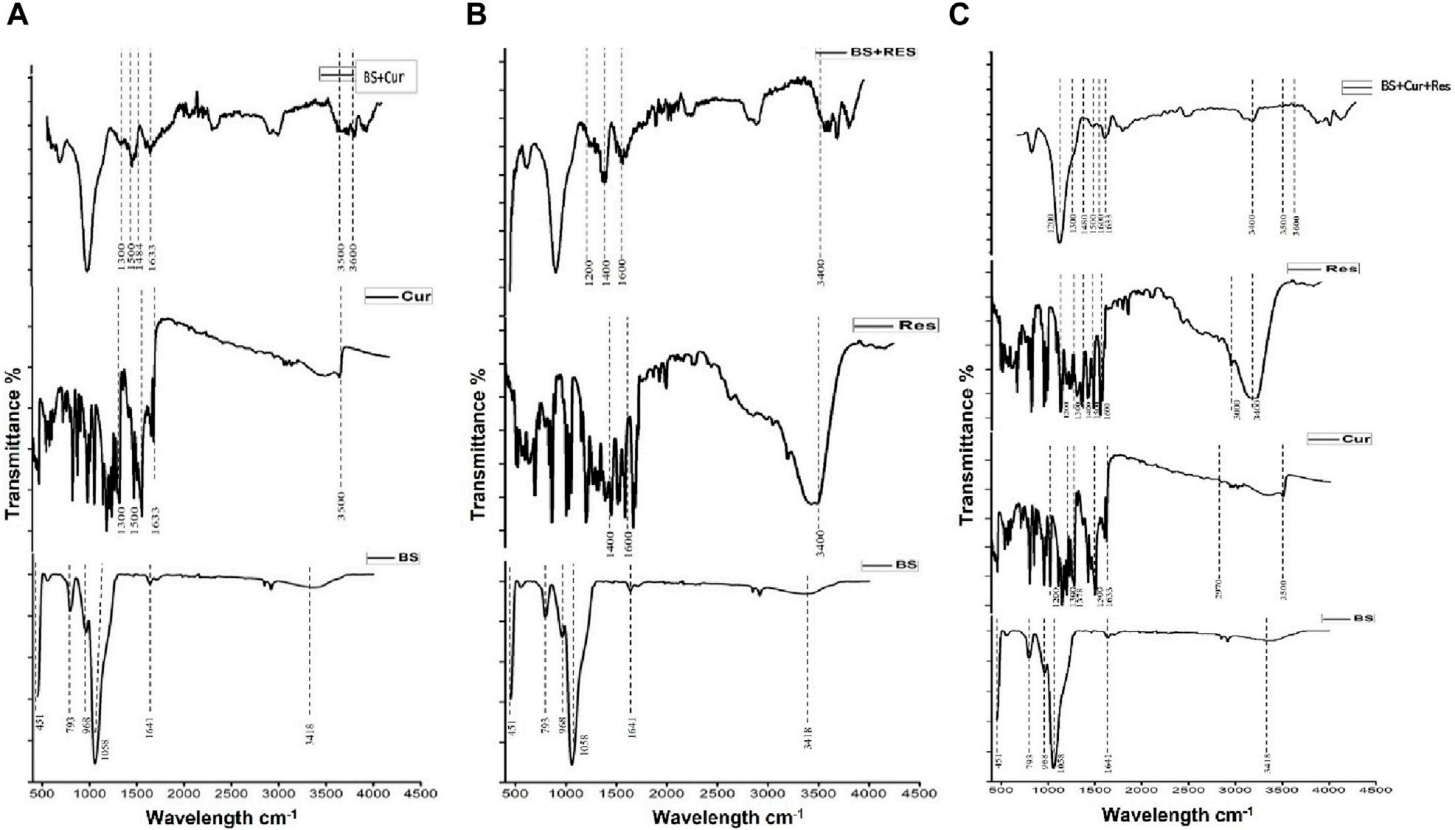
**(A)** FTIR spectrum of curcumin loaded BS. **(B)** FTIR spectra of resveratrol loaded BS. **(C)** FTIR spectra of curcumin and resveratrol loaded BS.

The FTIR spectrum (450–4,000 cm^−1^) of the BS extracted from *Equisetum myriochaetum* is illustrated in Figure 2. The analysis confirms the presence of BS. The prominent peak at 1,058 cm^−1^ corresponds to Si-O-Si stretching, while the band at 451 cm^−1^ indicates Si-O out-of-plane deformation. Moreover, the sharp peak at 793 cm^−1^ represents Si-O bending, and these three peaks are the most distinctive for BS presence. The peak at 968 cm^−1^ is linked to Si-OH stretching, the minor peak at 1,641 cm^−1^ signifies C-O bending, and the broad peak at 3,418 cm^−1^ is associated with O-H stretching (Shokri et al., 2009).

FTIR analysis also validated the successful preparation of Cur-BS, Res-BS, and Cur-Res-BS. In Figure 2, distinct curcumin bands are evident in both the curcumin graph and BS + Curcumin graph. The strong peak at 3,500 cm^−1^ corresponds to the stretching vibration of hydrogen-bonded O-H groups, while a small peak at 2,970 cm^−1^ corresponds to the asymmetric CH stretching of alkanes. The peak at 1,633 cm^−1^ represents the C-H frequency of the aromatic ring stretching. Bands observed at 1,500 cm^−1^ correspond to the conjugated carbonyl stretching, C = O and C = C vibration, while the peak at 1,300 cm^−1^ indicates the aromatic stretching vibrations of the benzene ring from the curcumin and the presence of these peaks in the BS + Curcumin spectra confirms the loading of curcumin onto the BS (Kotcherlakota et al., 2016). Also, the band at 1,378 cm^−1^ represents the CH_3_ bend, and the peak at 1,200 cm^−1^ corresponds to the C-O stretching of the carboxylic group (Gumireddy et al., 2019; Elbialy et al., 2020; Hettiarachchi et al., 2021). The Cur-BS spectrum reveals broad peaks at 3,500 and 3,600 cm^−1^, attributed to the asymmetric and symmetric stretching modes of N-H of APTES. Peaks at 1,562 and 1,484 cm^−1^ correspond to NH_2_ deformation modes of the amine groups, confirming amino-functionalization (Majoul et al., 2015).

On the other hand, both FTIR spectra of only Res and Res-BS show distinct resveratrol bands. A broad peak at 3,400 cm^−1^ corresponds to the phenolic O-H stretching vibration, while the peak at 300 cm^−1,^ which corresponds to the CH stretching of aromatic groups, indicates that the resveratrol has been immobilized successfully (Zuo and Ma, 2024). Moreover, peaks between 1,400 and 1,600 cm^−1^ represent the C-C aromatic double bond stretching vibrations. Also, the peak at 1,300 cm^−1^ indicates the C=C stretching of the ketone group, and the peak at 1,200 cm^−1^ corresponds to the C-O stretching of a carboxylic group (Gumireddy et al., 2019; Bhatt et al., 2020; Lin et al., 2021). Additionally, in the Res-BS spectrum, there are bands representing the formation of amide bonds between APTES and GTA, located between 1,200 and 1,600 cm^−1^ (Radhakrishnan et al., 2014). The IR spectrum of Cur-Res-BS displays a blend of distinctive peaks from both curcumin and resveratrol. Consequently, specific peaks may overlap between the two compounds, such as the bands ranging from 3,219 to 3,328 cm^−1^, found in both curcumin and resveratrol, representing OH stretching. Additionally, the bands at 1,600 cm^−1^ in both compounds indicate the C=C stretching of aromatic rings (Gumireddy et al., 2019).

### 3.3 Determination of weight loss by thermogravimetric analysis (TGA) and XRD analysis of the BS

**FIGURE 3:**
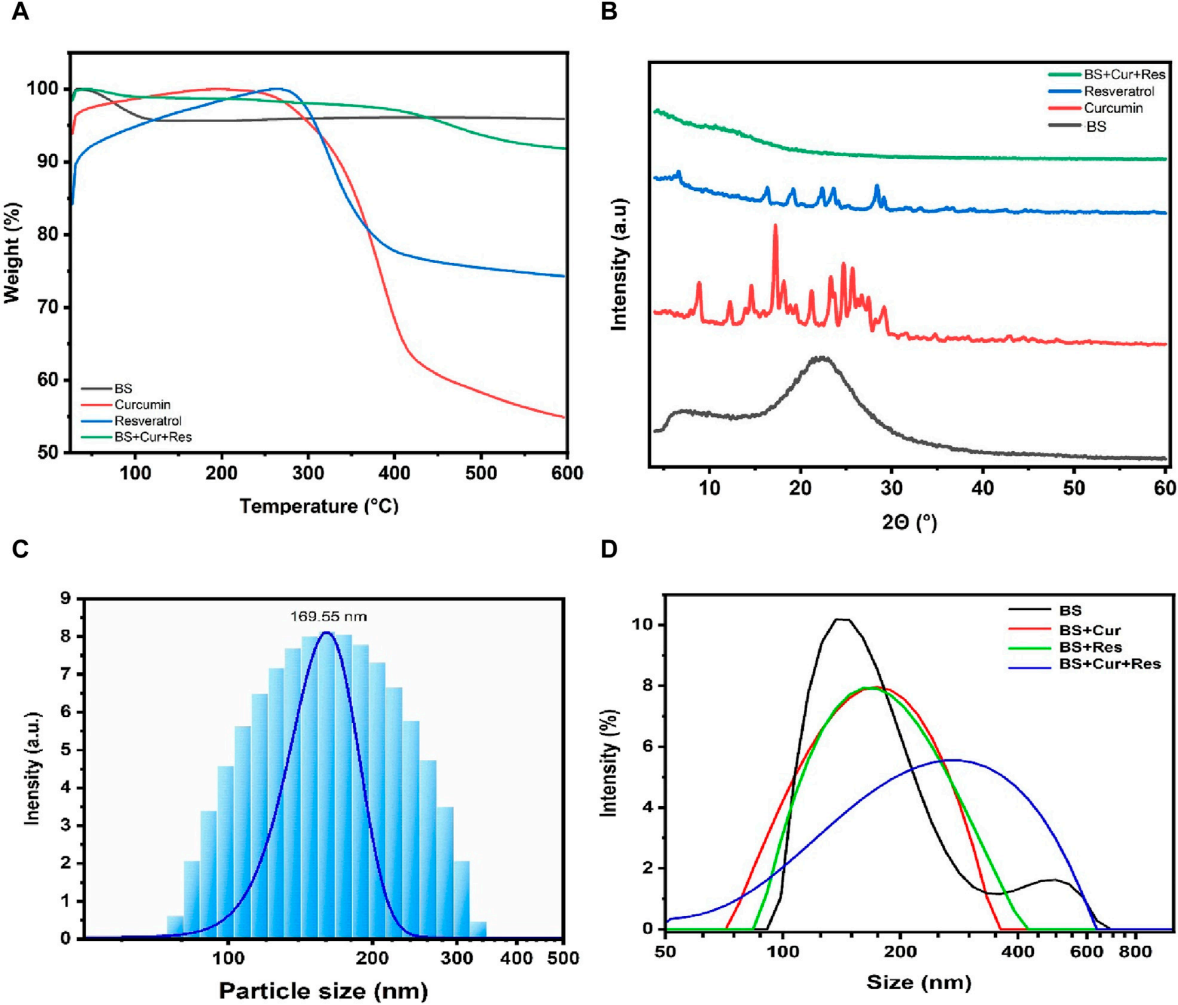
**(A)** TGA thermograms. **(B)** XRD diffractograms of BS, pure curcumin, pure resveratrol and immobilized bioformulation (BS + Cur + Res). **(C)** DLS measurement of BS. **(D)** Variations in the particle size of BS post immobilization with curcumin, resveratrol and the bioformulation (BS + Cur + Res).

The relationship between mass loss and temperature for the BS, curcumin, resveratrol and the bioformulation (BS + Cur + Res) was depicted in a graph (Figure 3A). The substantial weight reduction observed at 100°C in the BS sample might be explained by the desorption of absorbed water on the BS surface, as suggested by Sabeela et al. (2019). Additionally, the weight loss between 300°C and 500°C could be attributed to the decomposition of organic groups (Lin and Zhou, 2015). Notably, the decrease in weight loss after 500°C suggests the successful attainment of pure BS (Choi et al., 2020). The peak of BS, suggests that its degradation transition starts approximately at 50°C and finishes around 125°C. The weight loss is about 4.3%, which could be due to the removal of moisture from the sample. In the curcumin peak, it is observed that the degradation step occurs approximately at 241°C-and finishes around 423°C and has a weight loss of 45.1%. From the resveratrol peak, the unique degradation step starts at 279°C and has a weight loss of approximately 25%. In the case of the immobilized bioformulation (BS + Cur + Res), two degradation processes have been noticed. The first step of degradation is imperceptible and occurs at the same temperature as the BS sample and the weight loss is 2.7%, suggesting that it originates from sample moisture. The second step begins at approximately 400°C, a temperature higher than those observed in curcumin and resveratrol, signifying that these compounds gained more thermal stability as they are covalently attached to the BS. A total of about 8.2% of the bioformulation’s weight has been lost. A comparison of the weight loss between BS and BS + Cur + Res inferred that approximately 3.9% of Cur + Res was incorporated into the bioformulation.

XRD analysis was performed to detect the presence of crystals, which can alter the solubility of the samples (Gou et al., 2021). Figure 3B displays the high-angle XRD diffractograms for BS, curcumin, resveratrol, and bioformulation (BS + Cur + Res). The XRD patterns of BS derived from *Equisetum myriochaetum* exhibited an absence of sharp diffraction peaks, signifying the absence of crystalline character in the samples. The diffraction pattern of BS samples displayed broad peaks centered at approximately 2*θ* = 22°, affirming the amorphous state of the BS samples. This analysis provides evidence of the presence of structured pores attributed to a consistent periodic variation in the electron density within the BS (Zhang et al., 2018). Regarding curcumin, there were several medium-strength diffraction peaks visible at 2ϴ angles 8.94, 9.33, 9.38, 18.31, 23.93, and 25.13°, 29.18; the maximum intensity diffraction peak for pure curcumin was observed at a 2ϴ angle 17.36° indicating the high crystalline nature. Likewise, for pure crystalline resveratrol, the high-intensity diffraction peaks showed up at 2ϴ angles of 6.7, 16.58, 19.46, 22.67, 23.88, and 28.5° (Gumireddy et al., 2019). However, when curcumin and resveratrol were immobilized within BS, diffraction peaks of pure curcumin and resveratrol were invisible, inferring that the crystalline character of pure curcumin and resveratrol is highly decreased. Since curcumin and resveratrol are primarily loaded into the pores of the amorphous BS, with only a small quantity located on the surface of the particles or in the interparticle gaps, therefore, the patterns of crystalline peaks for curcumin and resveratrol are diminished. Conversely, an extra diffraction peak appeared at 2ϴ angle 14.11°, which could be due to polymorphic changes in the phytochemicals.

### 3.4 Zeta potential and particle size measurement

The average particle size and the zeta potential of the extracted BS were found to be 170 nm and −37.8 mV, respectively as shown in Figure 3C. A higher positive or negative zeta potential value (+30 or −30 mV) prevents the agglomeration of the particles (Zając et al., 2023). BS exhibited a value higher than −30 mV, which indicates that these BS particles are electrostatically stable and the higher negative value also suggests that the BS has a higher surface area, which is also confirmed through BET analysis. The particle size distribution ranges from 77 to 222 nm; the fine and coarse particle distribution was almost negligible, while the range of particle size distribution was found to be high, as confirmed by the polydispersity index (PDI 20.17). Also, we observed shifts in the peak of BS when curcumin, resveratrol and curcumin + resveratrol were immobilized onto the BS, as depicted in Figure 3D. Additionally, we measured the zeta potential of samples after immobilization of curcumin, resveratrol and curcumin-resveratrol and the changes in the zeta potential values confirm the attachment of the phytochemicals onto the BS.

### 3.5 Surface area determination by the Brunauer–Emmett–Teller (BET) method

Two different BS samples (obtained from branches and stems) were analyzed with the BET method to determine the surface area, pore volume, and pore diameter. The nitrogen adsorption pore size distribution curves suggested that the BS obtained from *Equisetum myriochaetum* branches exhibit a surface area of 318.266 m^2^/g, a pore volume of 0.625 cc/g, and a pore diameter of 4.918 nm. Similarly, BS obtained from *Equisetum myriochaetum* stems demonstrates a surface area of 364.15 m^2^/g, a pore volume of 0.693 cc/g, and a pore diameter of 4.926 nm. Furthermore, the analysis revealed an isotherm of type IV, a classification commonly employed for the characterization of mesoporous solids (Yurdakal et al., 2019).

### 3.6 Scanning electron microscope (SEM) imaging

**FIGURE 4:**
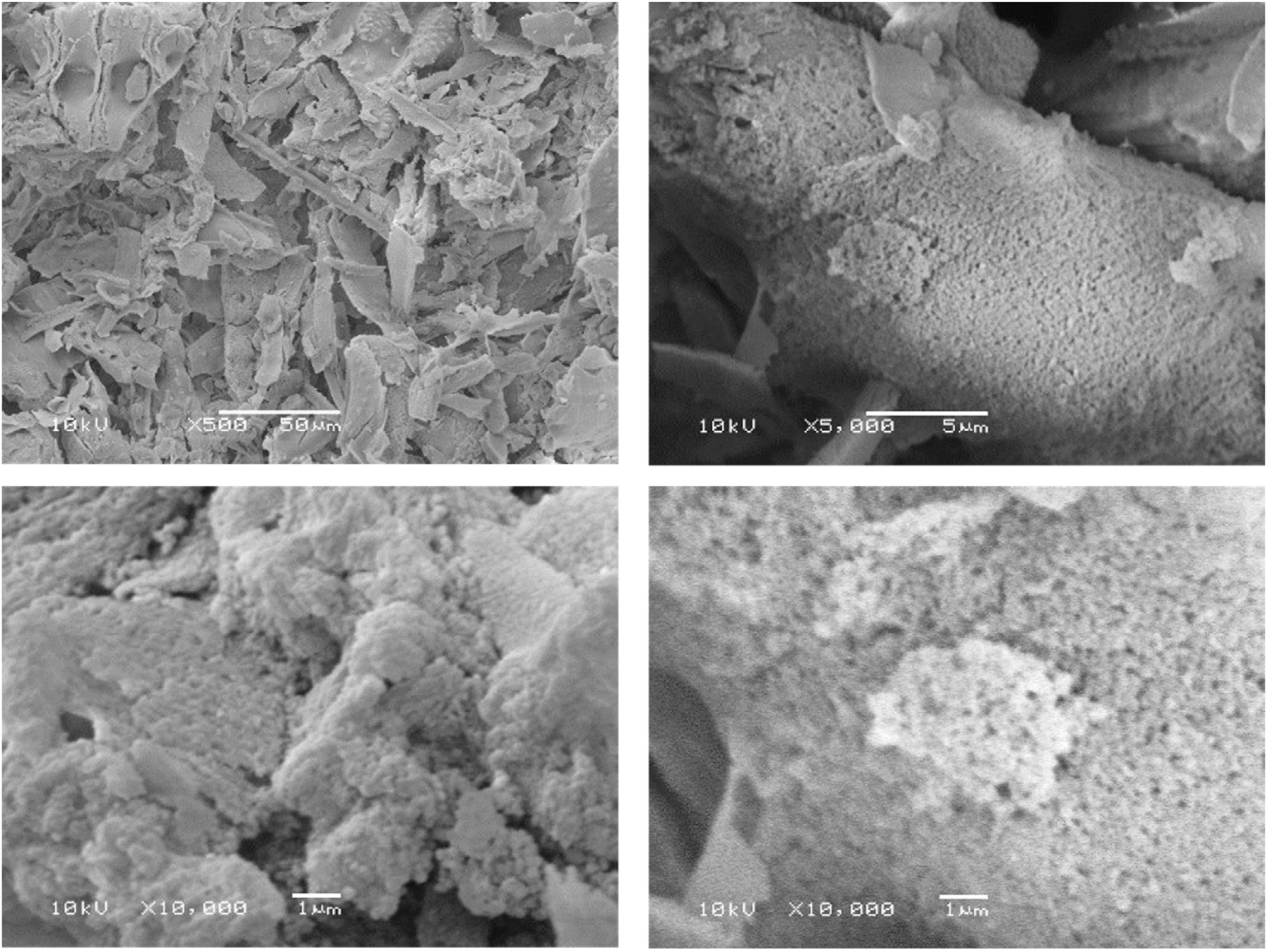
SEM to examine the surface morphology of BS.

SEM analysis was conducted to examine the topographic surface of BS, as depicted in Figure 4. The SEM images were captured at magnifications of 50, 5, and 1 μm, confirming the porous nature of the material and establishing a consistent porous morphology. Nevertheless, exploring at a higher magnification of 200 nm could offer precise pore size details.

### 3.7 Determination of encapsulation efficiency by UV-visible

The encapsulation efficiency of the current three bioformulations was established by employing a calibration curve for both curcumin and resveratrol. To elaborate, the Cur-BS contained roughly 36.32% of curcumin enclosed within their pores, the Res-BS encapsulated approximately 56.5% of resveratrol in their pores, and the Cur-Res-BS held about 45% of curcumin and 64% of resveratrol loaded in their pores.

### 3.8 Phytochemicals (curcumin and resveratrol) release kinetics

**FIGURE 5:**
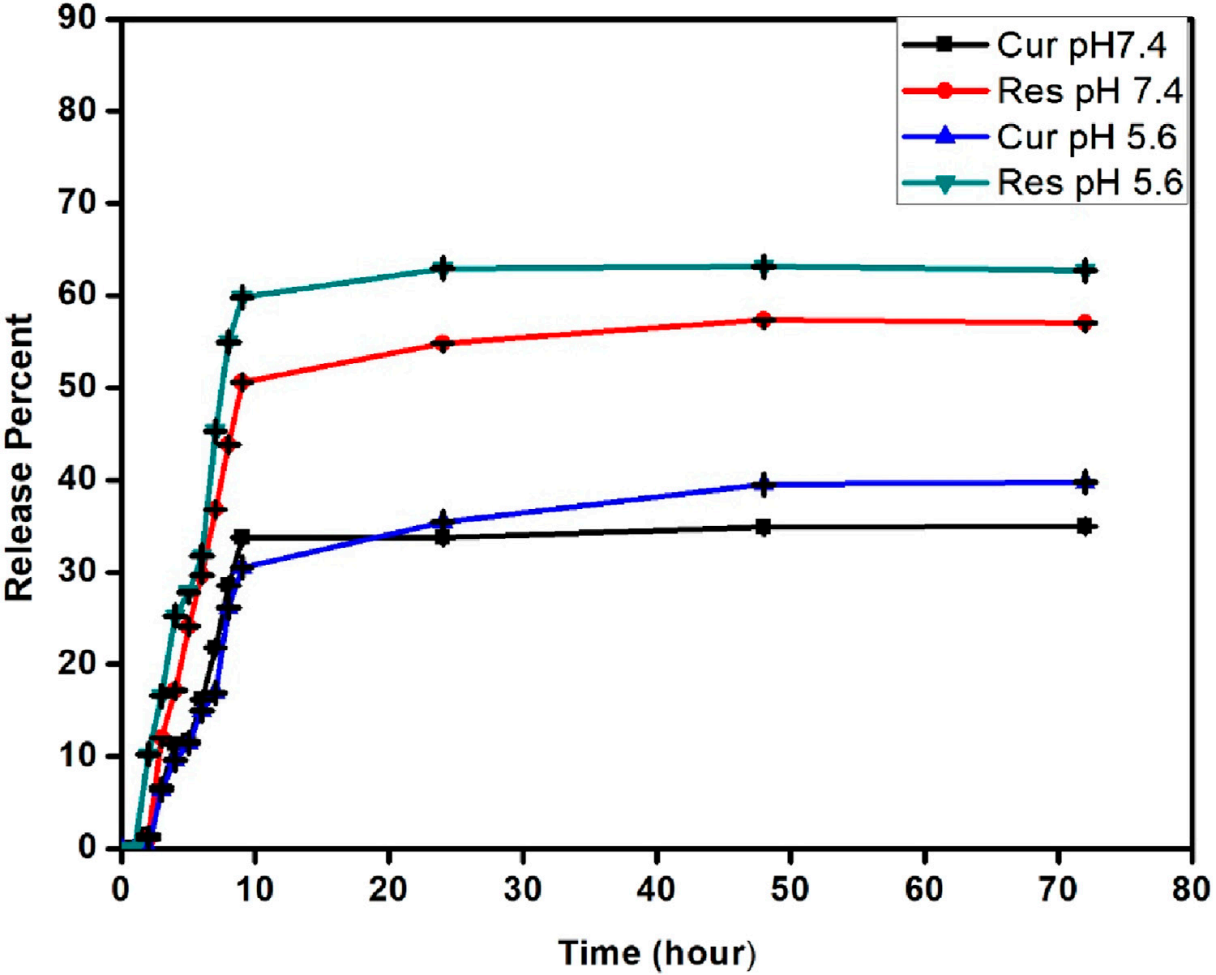
Phytochemicals (Curcumin and Resveratrol) release kinetics.

The *in vitro* phytochemicals release profile of Cur - Res loaded BS was spectrometrically obtained at different time intervals (0, 1, 2, 3, 4, 5, 6, 7, 8, 9, 24, 48 and 72 h) is shown in Figure 5. The release study was conducted using phosphate buffer of two different pH; pH 7.4, which represents the physiological pH of the human body, and pH 5.6, which reflects the pH of the cancer cell microenvironment at 37°C. The cumulative release rate of both the phytochemicals was found to be slow and sustained over the period of 72 h with a higher release percent of resveratrol in comparison to curcumin at both pHs. Additionally, at the lower pH (5.6), there was a somewhat higher release of both phytochemicals with 30.97% of curcumin and 60.39% of resveratrol compared to 20.83% of curcumin and 50.39% of resveratrol at pH 7.4. This experiment simulated at different physiological pH demonstrated that only a small percentage of phytochemicals was released for both curcumin and resveratrol after 72 h of vigorous stirring. The negative charge of curcumin and the strong positive charge of silanized BS and the crosslinking of resveratrol with glutaraldehyde can contribute towards the slow and sustained release of these phytochemicals.

### 3.9 Cell viability assay of HCT-116 and CACO-2 cells

**FIGURE 6:**
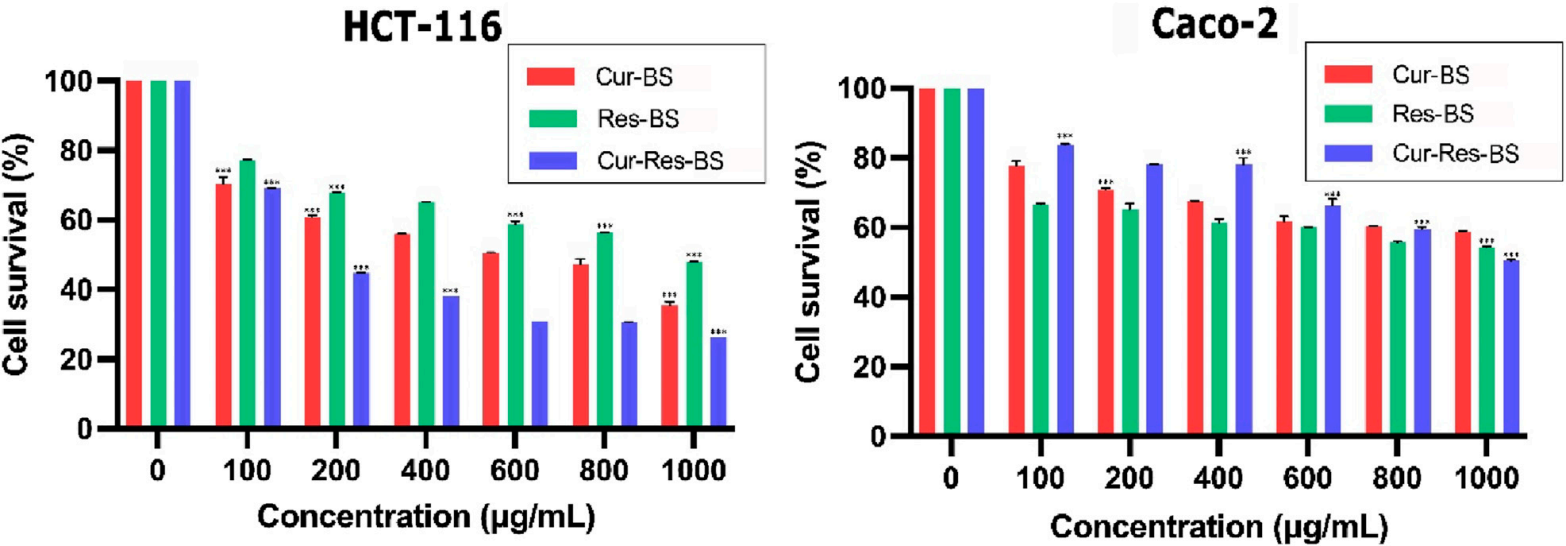
Comparison between the three different bioformulations (Cur-BS, Res-BS, and Cur-Res-BS) on HCT-116 and Caco-2 cells after 24 h of treatment. ***p < 0.001.

**FIGURE 7:**
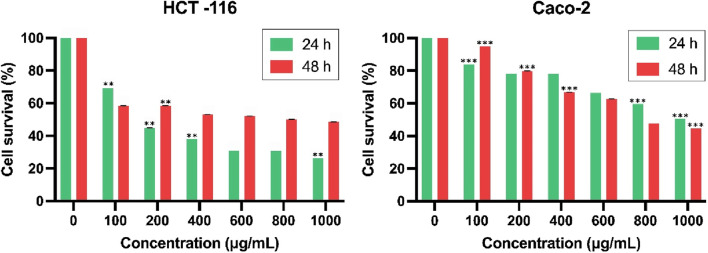
Comparison between combined treatment Cur-Res-BS on HCT-116 and Caco-2 cells at 24 and 48 h **p < 0.01, and ***p < 0.001.

The present study investigated the synergistic effects of nanoformulated curcumin and resveratrol on cell viability *in vitro*, utilizing two CRC cell lines: HCT-116 and Caco-2. The MTT assay was performed with bioformulations concentrations ranging from 100 to 1,000 μg/mL over 24 and 48 h, while untreated cells were used as the control group. In the case of HCT-116 cells treated with Cur-BS, Res-BS, and Cur-Res-BS, a significant reduction in cellular viability was noted after 24 h. The IC_50_ dose values were approximately 624.878, 470.7, and 380.84 μg/mL, respectively (Figure 6). Notably, the combined bioformulation (Cur-Res-BS) exhibited the most pronounced effect on cell growth, demonstrating a cell viability of 26.32% at 1,000 μg/mL. Further evaluation at 48 h revealed an IC_50_ value of 741.637 μg/mL and a cell viability of 48.48% at 1,000 μg/mL for the combined bioformulation (Figure 7). Caco-2 cell viability was assessed after 24 h treatment with the three different bioformulations. The resulting IC_50_ values were approximately 1,321.77, 1,136.27, and 1,002.42 μg/mL, respectively (Figure 6).

Despite the Caco-2 cell line’s varied response, requiring higher concentrations to achieve the IC_50_ dose, the combined treatment displayed a substantial impact on cell growth, yielding cell viability of 50.50% at 1,000 μg/mL. Moreover, the effect of the Cur-Res-BS nanoformulation on Caco-2 cells after 48 h resulted in an IC_50_ value of 846.171 μg/mL and a cell viability of 44.7% at 1,000 μg/mL (Figure 7). Furthermore, HCT-116 cells exhibited a more significant reduction in cell growth when exposed to the Cur-Res-BS bioformulation for 24 h. This difference was evident in the IC_50_ values, with HCT-116 cells showing an IC_50_ of 380.84 μg/mL, whereas Caco-2 cells had an IC_50_ of 1,136.27 μg/mL at 24 h.

### 3.10 Cytotoxicity assays of active substances on HCT-116 and Caco-2 cells

**FIGURE 8:**
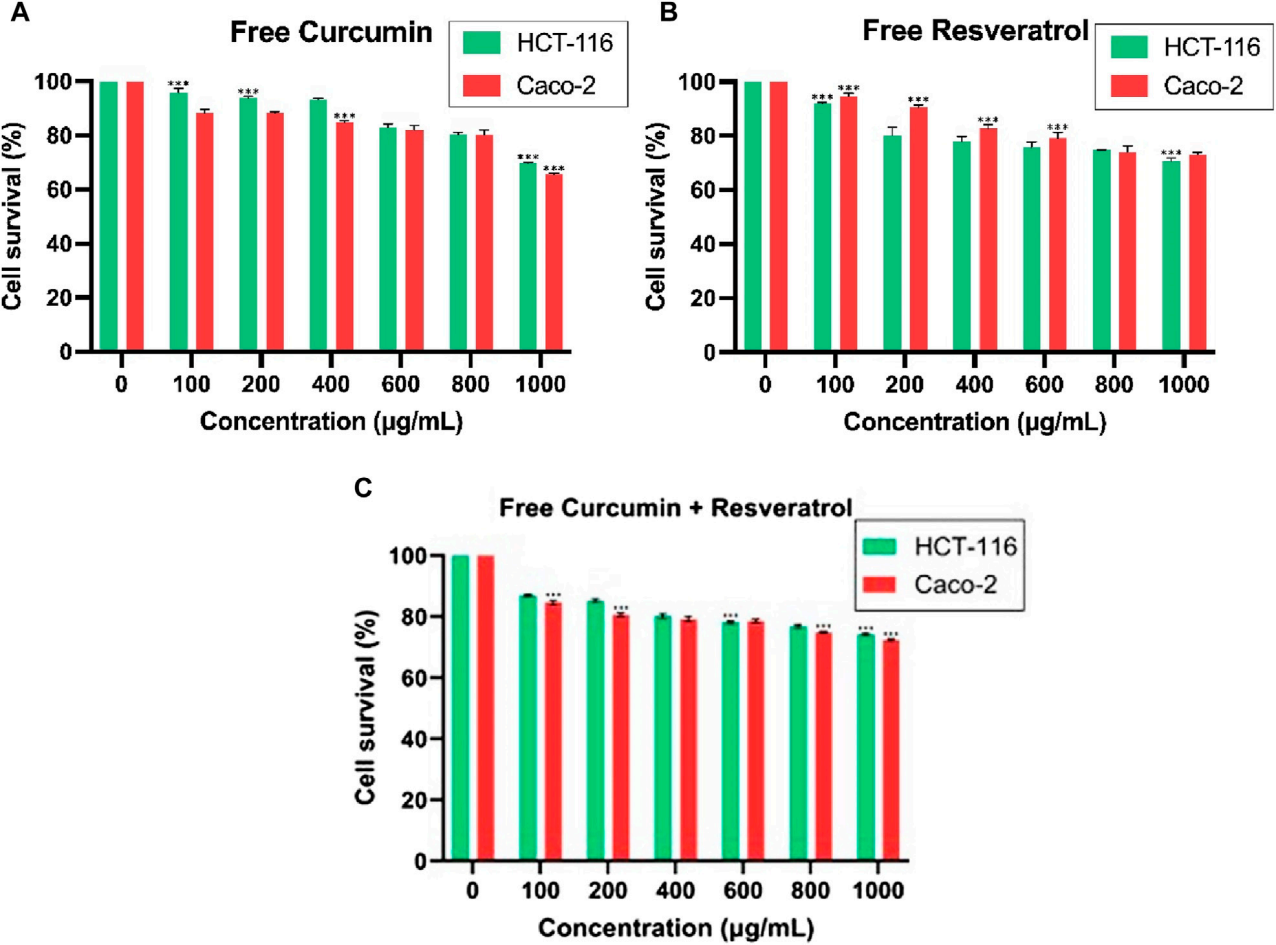
Cytotoxic effect of **(A)** free Curcumin and **(B)** free Resveratrol **(C)** free Cur-Res on HCT-116 and Caco-2 for 24 h incubation time. ***p < 0.001.

These outcomes were also compared with cytotoxicity assays conducted using concentrations ranging from 100 to 1,000 μg/mL of free curcumin and free resveratrol and a combination of free curcumin and resveratrol on both CRC cell lines. Free curcumin, free resveratrol, and also the combination of curcumin + resveratrol did not induce such a marked level of cytotoxicity as compared to the bioformulations (Figure 8). After 24 h of exposure to free curcumin, cell viability was 69.8% for HCT-116% and 65.62% for Caco-2 cells at 1,000 μg/mL, while, after 24 h of free resveratrol exposure, cell viability was 70.67% for HCT-116% and 73.09% for Caco-2 cells at 1,000 μg/mL. Similarly, the free Curcumin-Resveratrol combination after 24 h of exposure displayed 74.2% cell viability for HCT-116% and 72.3% for Caco-2 at 1,000 μg/mL. One possible reason for getting the higher values even at higher concentrations of free Curcumin-Resveratrol could be the increased formation of formazan by resveratrol in the reaction mixture, which has been previously reported to interfere in some cases with the MTT assay (Siddiqui et al., 2011; Stepanenko and Dmitrenko, 2015).

### 3.11 Cytotoxicity assay of BS on HCT-116 and Caco-2 cells

**FIGURE 9:**
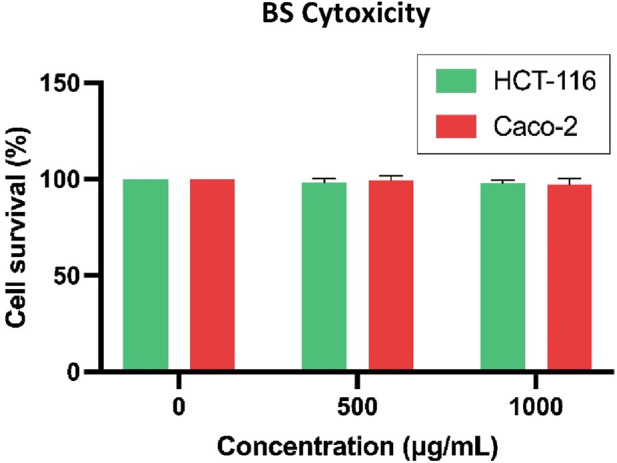
Cytotoxicity of BS on HCT-116 and Caco-2 after 24 h of incubation time.

The effect of BS was assessed on HCT-116 and Caco-2 cell lines at concentrations of 500 and 1,000 μg/mL, resulting in cell viability of 98.41% at 500 μg/mL and 97.92% at 1,000 μg/mL for HCT-116 cells. Additionally, cell viability was observed to be 99.68% at 500 μg/mL and 97.34% at 1,000 μg/mL for Caco-2 cells (Figure 9).

### 3.12 Cytotoxicity assay of Cur-Res-BS on HEK-293 cell line and selectivity index calculation

**FIGURE 10:**
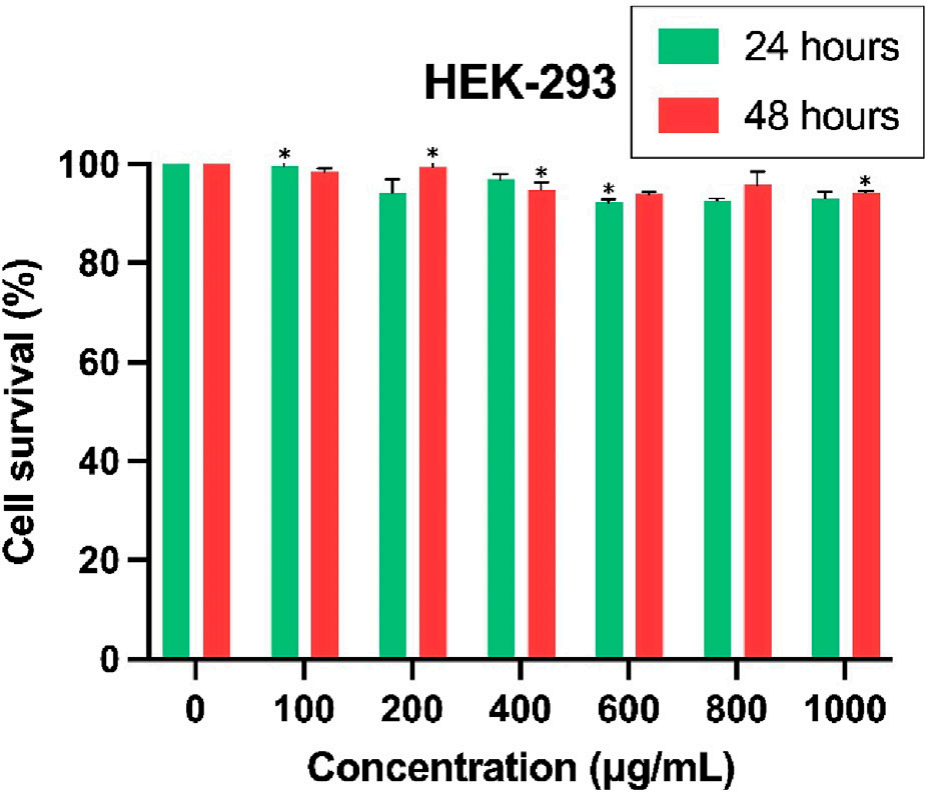
Evaluation of the cytotoxic effects of Cur-Res-BS on the HEK-293 cell line after 24 and 48 h of exposure. *p < 0.05.

The evaluation of Cur-Res-BS on HEK-293 cells was conducted after 24 and 48 h of treatment to confirm the bioformulation’s limited cytotoxicity on normal cells. IC_50_ values of 6,766.94 and 8,625.61 μg/mL were determined after 24 and 48 h of incubation, respectively, as depicted in Figure 10.

Subsequent to this, the SI was determined to underscore the bioformulation’s specificity towards cancer cells. The IC_50_ values of 380.84 and 6,766.94 μg/mL for HCT-116 and HEK-293 cell lines, respectively, were employed in the computation of the SI, resulting in a value of 17.77.

### 3.13 Gene expression analysis by qPCR

**FIGURE 11:**
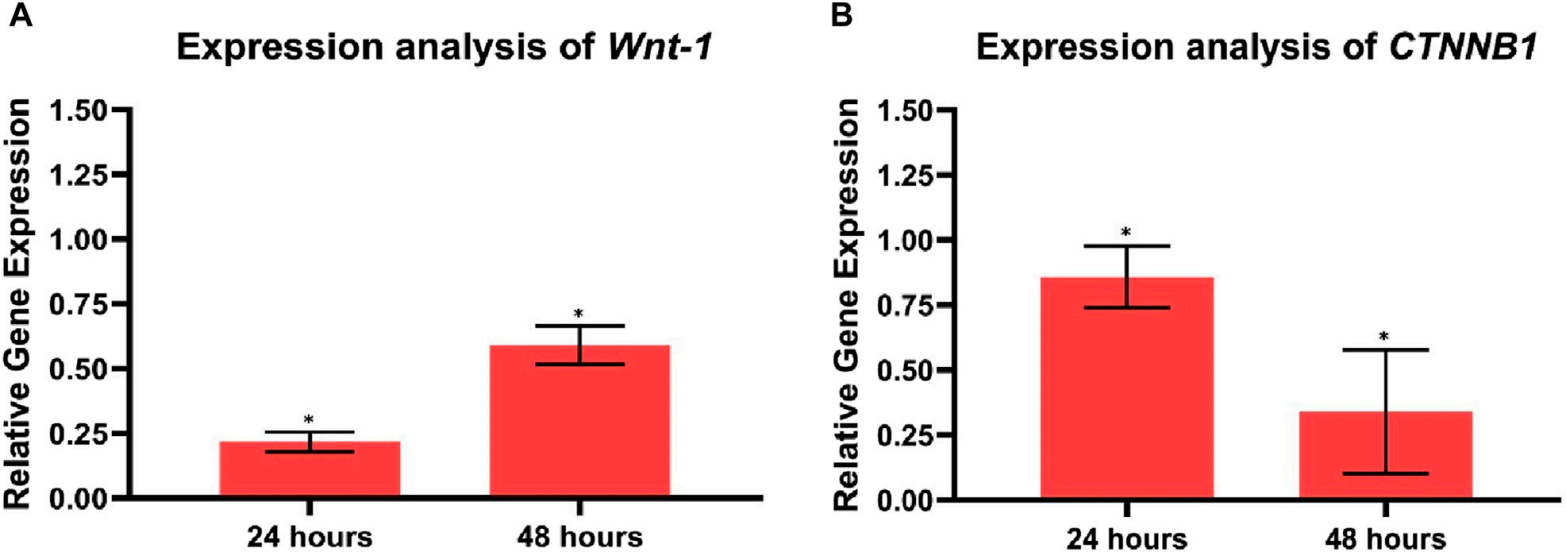
Analysis of proliferation-related gene expression **(A)** *Wnt-1*, **(B)** *CTNNB1* in control, and HCT-116 treated cells after 24 and 48 h of incubation. Each bar graph represents the mean values of relative fold change ± standard deviation obtained from triplicate assays. *p < 0.05.

The Cur-Res-BS bioformulation demonstrated significantly reduced cell viability in HCT-116 cells, prompting its selection for RNA extraction and subsequent analysis of proto-onco- and tumor suppressor genes (*TP53*, *Bax*, *Wnt-1*, and *CTNNB1*). Cells were exposed to the experimental IC_50_ value of the combined bioformulation (380.84 μg/mL) for 24 and 48 h, and the influence of the bioformulation and the interaction between bioformulation and time were evaluated. As shown in Figure 11, the study unveiled there was a significant downregulation of *Wnt-1* by 0.21-fold and 0.59-fold after 24 and 48 h of treatment, respectively (*p* < 0.05); however, the interaction between the bioformulation and time was not statistically significant (*p* > 0.05). Moreover, it significantly decreased the expression of *CTNNB1* by 0.85-fold and 0.34-fold after 24 and 48 h of treatment, respectively (*p* < 0.05), while the interaction between the treatment and time was also statistically significant (*p* < 0.05).

**FIGURE 12:**
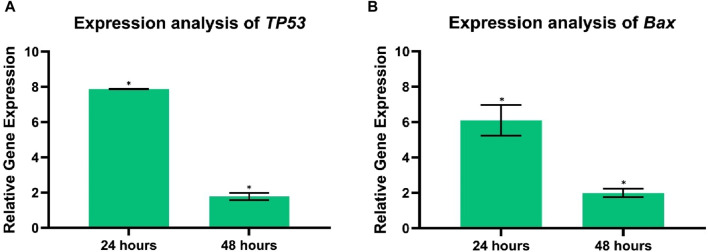
Analysis of tumor suppressor gene and apoptosis-related gene expression **(A)** *TP53*, **(B)** *Bax* in control, and HCT-116 treated cells after 24 and 48 h of incubation time. Each bar graph represents the mean values of relative fold change ± standard deviation obtained from triplicate assays. *p < 0.05.

Additionally, after 24 and 48 h of treatment exposure, the Cur-Res-BS bioformulation significantly upregulated *TP53* by 7.87-fold and 1.77-fold, respectively (*p* < 0.05), while the interaction between the bioformulation and time was also statistically significant (*p* < 0.05). Additionally, the *Bax* expression also significantly increased by 6.1-fold and 1.99-fold after 24 and 48 h of exposure, respectively (*p* < 0.05), as shown in Figure 12. Nevertheless, the interaction between the bioformulation and time was not statistically significant (*p* > 0.05).

## 4 Discussion

CRC stands as a significant challenge for global healthcare systems, ranking as the third most frequently diagnosed cancer worldwide and representing the second leading cause of cancer-related deaths, affecting both men and women (Lewandowska et al., 2022; Osorio-Pérez et al., 2023). Consequently, there is a dedicated focus on innovative approaches to cancer treatment, exploring new strategies and solutions (Biller and Schrag, 2021). While conventional methods like chemotherapy and radiotherapy have historically been crucial in cancer treatment, their associated side effects significantly impact patients’ quality of life (Khan et al., 2019). As a result, recent scientific attention has shifted towards natural remedies, particularly investigating the potential of natural phytochemicals and biocompatible bioformulations (Berretta et al., 2020). Studies have highlighted the ability of curcumin and resveratrol to significantly inhibit proliferation and induce apoptosis in various cancer cells, establishing them as vital components in cancer therapy (Vervandier-Fasseur and Latruffe, 2019; Zheng et al., 2022).

In this study, BS was extracted from *Equisetum myriochaetum* and the subsequent surface modification involved the immobilization of APTES, a widely employed silane coupling agent, introducing an amine-terminated group through silanization. This procedure established a covalent bond between the surface of BS and curcumin (Majoul et al., 2015). Following this, the silanized samples were subjected to immobilization with GTA, incorporating an aldehyde group to facilitate the covalent binding of resveratrol (Ionescu, 2022). These chemical modifications enabled the immobilization of curcumin and resveratrol onto amine-terminated and aldehyde groups, respectively, leading to the creation of three distinct bioformulations: Cur-BS, Res-BS, and Cur-Res-BS.

Thorough characterization of bioformulations is essential for a comprehensive understanding of their composition, as well as their physical, optical, and morphological attributes. This study employed specific characterization techniques to scrutinize these facets, including FTIR, TGA, SEM, XRD, BET, and UV-visible spectrophotometry. FTIR was employed to assess the purity of BS extracted from *Equisetum myriochaetum*. Noteworthy peaks observed in the BS spectrum were identified at 451, 793, 968, 1,058, 1,641, and 3,418 cm^−1^, corresponding to characteristic stretching and vibrational bonds between atoms. These peaks indicate the exclusive presence of BS, devoid of any detrimental elements (Shokri et al., 2009). Additionally, FTIR was used to confirm the successful immobilization of curcumin and resveratrol through APTES and GTA functionalization. Distinct peaks observed in the FTIR spectra of Cur-BS, Res-BS, and Cur-Res-BS, including those at 1,058, 1,400, 1,550, 1,600, 3,000, 3,300, 3,500, and 3,600 cm^−1^, are attributed to the presence of specific functional groups, thus confirming the success of surface modification process (Majoul et al., 2015; Elbialy et al., 2020). XRD analysis indicated the amorphous nature of the BS samples, positively influencing their solubility. The abolition of crystalline peaks of curcumin and resveratrol makes the bioformulation compatible, and the appearance of a peak around 2ϴ angle 14.11° confirms the successful attachment of curcumin and resveratrol onto the BS. The same was also confirmed by DLS and TGA studies. Furthermore, SEM delineated the porous surface nature of the BS, a characteristic corroborated by BET analysis, which affirmed the existence of pores featuring an average diameter of 4.9175 nm and an average volume of 0.652 cc/g.

The assessment of encapsulation efficiency is crucial to ascertain the successful immobilization of therapeutic agents within the inert silica host (Kankala et al., 2022). Our investigation revealed that approximately 45% of curcumin and 64% of resveratrol were effectively loaded within the pores of the Cur-Res-BS bioformulation. It is pertinent to acknowledge the diverse encapsulation efficiencies observed across different nanocarriers in prior research endeavors. For instance, solid lipid nanoparticles (SLN) exhibited encapsulation efficiencies of 74.42% for curcumin and 79.84% for resveratrol (Gumireddy, 2017), while zein-based solid nanocarriers demonstrated 54% encapsulation for curcumin and 71% for resveratrol (Leena et al., 2022). Additionally, co-loaded conjugated polymer achieved an impressive efficiency of 91.3% for curcumin and 83.2% for resveratrol (Zheng et al., 2022). Although our study did not attain the anticipated high encapsulation efficiency, it is crucial to recognize that similar challenges have been encountered by fellow researchers in bioformulation studies. Several factors could underlie the observed reduction in encapsulation efficiency. Firstly, the swift release of therapeutic cargo during the loading process could be attributed to weak interactions between the surface of BS and the guest molecules (Kankala et al., 2022). Additionally, it has been observed that a significant portion of phytochemical compounds may adhere to the external surface of BS rather than being restricted within the interior pores. This phenomenon is linked to the obstruction of silica pore gates, resulting in a decline in encapsulation efficiency (Asgari et al., 2021). Furthermore, in-depth characterization techniques and meticulous analyses are imperative to gain a comprehensive understanding of the encapsulation process, identifying potential avenues for enhancements in future studies.

APTES modification introduces amino groups onto the surface of BS, which can interact with drugs and affect their release profiles. For example, in the case of mesoporous silica nps (MSNs), the APTES-modified surface can significantly increase drug loading and decrease the rate of drug delivery (Ahmadi et al., 2014). Drug release studies have shown that APTES-modified pSi can release hydrophobic drugs like CPT continuously over extended periods, demonstrating the material’s stability and suitability for sustained drug delivery (Zhang et al., 2019). Glutaraldehyde is used to cross-link pectin-based delivery vehicles for the encapsulation of resveratrol; the cross-linking process is essential for creating nps that can specifically deliver resveratrol to the colon (Chung et al., 2020). The concentration of glutaraldehyde is a critical factor that affects both the cross-linking process and the encapsulation efficiency of resveratrol. The drug release profile at both pHs exhibits a slow and sustained release, where both the phytochemicals were released at a steady state. In the initial hours, resveratrol was released first, and thereafter release of curcumin was observed, which is in accordance with the immobilization protocol followed where the inner layer is supposed to be formed of curcumin and the outer layer is composed of resveratrol. The porous structure of the BS allows for the controlled release of curcumin, which can be designed to occur over extended periods. This gradual pattern of release could be advantageous as it can maintain the therapeutic window for a longer time period.

In recent studies, researchers have discovered that the combination of therapeutic agents such as curcumin and resveratrol enhances cytotoxicity and augments the effects of chemotherapy. However, to overcome the limitations associated with these compounds, including weak chemical stability, low solubility, and poor bioavailability, co-loaded bioformulations have been explored (Selvam et al., 2019). In our current study, we explored the cytotoxic effects of the Cur-Res-BS bioformulation on colorectal cancer cells, conducting viability assays on both HCT-116 and Caco-2 cell lines. The results demonstrated a significant reduction in cell viability, indicating a dose-dependent relationship. Specifically, the cytotoxic effects of Cur-Res-BS were observed in HCT-116 cells within the concentration range of 100–1,000 μg/mL, with an IC_50_ value of 380.84 μg/mL.

To the best of our knowledge, none of the studies have been carried out so far to demonstrate the cytotoxic effects of curcumin or resveratrol-loaded BS on colorectal cancer cells. Predominantly, existing studies on curcumin OR resveratrol-loaded BS focus on their cytotoxic impact on breast cancer cells, although a subset of analogous studies provides a comparative framework. Colloidal BS has been documented to enhance the biological efficacy of resveratrol on HT-29 colon cancer cells, exhibiting dose-dependent reductions and a 36% decline in cell viability at a concentration of 400 μM (Summerlin et al., 2016). Furthermore, investigations involving 5-fluorouracil, a widely used clinical chemotherapeutic agent, and curcumin-loaded BS revealed an IC_50_ of 21.3 μg/mL in Hep-2 laryngeal squamous cancer cells, emphasizing heightened synergistic effects (Wang et al., 2020). Conversely, examinations into the synergistic effect of curcumin and resveratrol in conjugated polymer nps on hepatocellular carcinoma cells reported an IC_50_ of 18.30 μM (Zheng et al., 2022).

Despite the relatively elevated concentrations employed in our study compared to analogous bioformulations assessed across diverse cancer cell types, it is imperative to underscore that the principal aim was to establish their efficacy and assess the expression of target genes as preliminary data. For ensuing research endeavors, exploration of lower concentrations is warranted to delineate the minimal thresholds eliciting maximal effects. This prudent approach will contribute to a more exhaustive comprehension of the nuanced responses of colorectal cancer cells to the proposed bioformulations. Conversely, the impact of Cur-Res-BS on Caco-2 cells was observed within the same concentration range. However, the cytotoxic impact observed in Caco-2 cells was not as pronounced as in HCT-116 cells. It is noteworthy that the IC_50_ value for Caco-2 cells was notably higher, measured at 1,002.42 μg/mL. This higher value indicates the need for elevated concentrations to achieve the IC_50_ dose response in this particular cell line. The discrepancy in the cytotoxic effects of Cur-Res-BS between HCT-116 and Caco-2 cells is remarkable. Further research is essential to comprehensively understand the bioformulation’s impact on Caco-2 cells. Several factors could contribute to the divergent outcomes in these two CRC cell lines. Genetic variations among cancer cell lines can influence the metabolism rates of compounds, the expression levels of detoxifying enzymes, and the efficiency of the multidrug resistance system in expelling cytotoxic agents, as demonstrated in prior studies (Santana-Gálvez et al., 2020). Furthermore, earlier research has indicated distinct responses of Caco-2 monolayers to resveratrol treatment, where cell growth inhibition was observed only when the treatment was administered apically, leaving the basolateral membrane unaffected (Polycarpou, 2013). Additionally, when subjected to identical treatments, HCT-116 and Caco-2 cells exhibited diverse responses, indicating the involvement of distinct mechanisms in these separate cell lines (Santana-Gálvez et al., 2020).

Additional cytotoxicity assays were conducted to assess the efficacy and safety of the bioformulations. Initially, the cytotoxicity assay of free curcumin, ranging from 100 to 1,000 μg/mL, resulted in high cell viability on both cell lines, with figures reaching 69.8% for HCT-116% and 65.62% for Caco-2 cells at 1,000 μg/mL. Similarly, the assay of free resveratrol yielded a cell viability of 70.67% for HCT-116% and 73.09% for Caco-2 cells. These outcomes indicate that the bioformulations exhibit a greater cytotoxic effect than the free phytochemicals on both cell lines at equivalent concentrations and conditions. The increased effectiveness of bioformulations in combating cancer, when compared to free phytochemical compounds, can be attributed to several factors. After initial interaction through ligand-receptor binding or non-specific interactions, the nps are typically internalized via endocytosis. The exact pathway of endocytosis can depend on the nanoparticle properties and cell type but often involves clathrin-mediated endocytosis, caveolin-mediated endocytosis, or macropinocytosis. The clathrin-mediated endocytotic pathway internalizes poly(lactic-co-glycolic acid), D,L-polylactide, poly(ethylene glycolco-lactide), and silica (SiO_2_)-based nanomaterials (Foroozandeh and Aziz, 2018). The average diameter, PDI, and size stability of nanocarrier formulations are among the parameters that determine whether or not they are appropriate for drug administration. The internalization process of the larger nps (≥165 nm) is through endocytosis but requires longer time and energy in comparison to the smaller nps (≤113 nm) (Xue et al., 2018). Cancer tissues often have leaky vasculature and poor lymphatic drainage, allowing larger particles to preferentially accumulate within the tumor tissue—a phenomenon known as the EPR effect. Tumor cells allow the entrance/exit of nanocarriers of a certain size (≤150 nm) through fenestrated capillaries (Danaei et al., 2018). It has also been reported that nps with sizes up to a few micrometers can also undergo endocytosis (Zhang et al., 2009). Though the PDI value of BS is high in our study, which indicates the existence of particles of different sizes, however, the size range is between ∼77 and ∼222 nm and as reported, particle size less than 200 nm can mediate their entrance through clathrin-mediated endocytosis while particle size above 200 nm are internalized through caveolae-mediated endocytosis. The process of macropinocytosis allows bacteria, viruses, apoptotic and necrotic cells, and antigen presentation to be swallowed. Micron-sized nps that are unable to enter into cells by the majority of other pathways can be internalized by this pathway (Foroozandeh and Aziz, 2018; Manzanares and Ceña, 2020). According to the values of zeta potential, the surface charges of BS changed from −37.8 to 2.3 mV when resveratrol was added and 2.7 mV when curcumin was added. A zeta potential value of 2.4 mV was obtained when both the phytochemicals were added to the BS, which implies that the addition of these phytochemicals imparts a positive charge to BS (Mohebian et al., 2023). As reported, positively charged nps are easily taken up by the negatively charged plasma membrane, leading to the disruption of the membrane integrity and can induce cell death. Additionally, positively charged nps have been demonstrated to produce a high level of intracellular ROS compared to neutral or negatively charged nps (Bhattacharjee et al., 2010). Furthermore, the enhanced impact of Cur-Res-BS, as opposed to free compounds, arises from the effectiveness of the bioformulation’s therapeutic window. This therapeutic window, which delineates the duration during which the treatment remains effective, is a crucial parameter for understanding the sustained efficacy of administered substances. In this context, a significant advantage of BS-based drug administration lies in the gradual release of drugs into cells, ensuring a prolonged period of effectiveness (Rosenholm et al., 2011).

Moreover, the results of the cell viability assay of BS on both cell lines showed minimal cytotoxicity, with cell viability measured at 97.92% at 1,000 μg/mL for HCT-116 cells and 97.34% at 1,000 μg/mL for Caco-2 cells. The disparity between these results and the cytotoxicity of the BS on the same cell lines is significant. These findings confirm the biocompatibility of the drug-free BS with both cell lines, as there is no substantial reduction in cell viability, indicating no safety concerns associated with the treatment in cellular terms. Previous studies have indicated that BS at 100 μg/mL also maintained viability above 80%, emphasizing the lack of apparent cytotoxicity on SW620 cells, indicating their biocompatibility (Liu et al., 2018). Additionally, it has been reported that drug-free BS do not exhibit obvious cytotoxicity to SW480 and NCM460 cells, asserting their biocompatibility and non-toxicity to cells, notably, only at extremely high concentrations of BS, around 25 mg/mL, exhibit cytotoxicity (Li et al., 2017). Furthermore, in the study conducted by Summerlin et al., 2016, it was observed that BS, when employed as a control, displayed negligible cytotoxicity in HT-29 and LS174T colon cancer cell lines. These findings are consistent with earlier research, providing further evidence of the non-toxic characteristics associated with BS.

The assessment of the cytotoxicity of the Cur-Res-BS formulation on the HEK-293 cell line revealed minimal toxicity, as evidenced by significantly elevated IC50 values of 6,766.94 μg/mL after 24 h of exposure and 8,625 μg/mL after 48 h of exposure, indicating low toxicity on healthy cells. The SI serves as a measure of the specificity of the bioformulation towards cancer cells compared to normal cells, with a higher SI value corresponding to greater selectivity (Veschi et al., 2020). The resulting SI of 17.77 signifies a substantial level of selectivity towards the HCT-116 cell line.

The process of cancer development unfolds through a series of sequential mutational events as the disease progresses (Ruiz-Manriquez et al., 2022). Crucial pathways like Wnt/β-catenin, p53, and Bcl-2 play pivotal roles in regulating various biological processes, including cell differentiation, proliferation, angiogenesis, apoptosis, and survival (Koveitypour et al., 2019). The human intestine, a highly dynamic organ, possesses remarkable regenerative capabilities, allowing it to replace its entire 7-meter-long cell lining weekly. This regenerative mechanism is primarily triggered by various stresses, including microbiological, chemical, and mechanical factors originating from digestion and evacuation processes (Beumer and Clevers, 2020). Numerous signaling pathways actively participate in the self-renewal, differentiation, and proliferation of cells. Considering the crucial function these pathways serve in cell renewal, it is expected that any changes in their elements can trigger pathological occurrences (Powell et al., 2011). It is essential to understand these pathways’ fundamental functions in both normal physiological states and under pathological conditions. Exploring the impact of naturally occurring phytochemicals that modulate these pathways in CRC is crucial to advancing our understanding and developing potential therapeutic interventions (Oliveira et al., 2022).

When genetic or epigenetic abnormalities induce mutations in the canonical Wnt/β-catenin signaling pathway, it leads to abnormal proliferation and tumor growth, particularly in CRC (Pashirzad et al., 2021). Elevated levels of *CTNNB1* and *Wnt-1* are detected in CRC patients compared to normal tissues (Siddiq et al., 2017). Previous research has indicated that curcumin and resveratrol exert a potent inhibitory effect on colorectal cancer cell proliferation by suppressing the Wnt/β-catenin signaling pathway (Cheng et al., 2019). Our results demonstrate a decrease in *Wnt-1* expression in HCT-116 of 0.21-fold after 24 h and 0.59-fold after 48 h (*p* < 0.05). However, the interaction between the bioformulation and time was not statistically significant (*p* > 0.05), therefore, more evidence is needed to prove that the effect of the bioformulation increases over time. Additionally, downregulation of *CTNNB1* expression in HCT-116 cells of 0.85-fold after 24 h and 0.34-fold after 48 h was observed (*p* < 0.05). Moreover, *Bax*, a crucial member of the Bcl-2 family and a regulator of the intrinsic apoptotic pathway, plays a pivotal role in inducing cell death (Kowalczyk et al., 2017). Furthermore, the *TP53* gene, frequently mutated in various cancers, including CRC, serves as a vital tumor suppressor gene (Slattery et al., 2019). Studies with the HCT-116 cell line have reported that curcumin-induced upregulation of *Bax* inhibits apoptosis (Moragoda et al., 2001). Additionally, the application of resveratrol has been reported to increase the expression of the tumor suppressor protein p53, which is crucial in regulating the cell cycle and apoptosis (Li et al., 2019). Exposure to Cur-Res-BS treatment resulted in a significant upregulation of *Bax* expression of 6.1-fold after 24 h and 1.99-fold after 48 h (*p* < 0.05). Nevertheless, the statistical analysis indicated that there was no significant interaction observed between the bioformulation and time (*p* > 0.05). Thus, further empirical evidence is required to substantiate that the impact of the bioformulation effect intensifies over time. Moreover, a significant upregulation of *TP53* expression levels of 7.87-fold after 24 h and 1.77-fold after 48 h, compared to the control group, was observed (*p* < 0.05).

## 5 Conclusion

In summary, this study marks the first attempt to explore the therapeutic effects of curcumin-resveratrol-loaded BS on CRC cells. Our results revealed a significant reduction of cancer cell viability following the administration of Cur-Res-BS when compared to the individual bioformulation, indicating a synergistic impact of the nanoformulated phytochemicals. Moreover, it was also observed that the anticarcinogenic effect of current bioformulations is much higher than the free phytochemical compounds, which implies a significant advantage of BS-based drug administration. Furthermore, comprehending the molecular changes brought about by the bioformulation in the pivotal genes is essential for laying the foundation for further stages of the investigation. Since the outcome of this study is promising, further research into the intricate mechanisms and prerequisites essential for the optimal functionality of this treatment is vital, including the associated risks and limitations. Last but not least, the development of an *in vivo* targeted delivery system of the current bioformulation would justify its real strength, and the current research would pave the way for that.

## Data Availability

The raw data supporting the conclusions of this article will be made available by the authors, without undue reservation.
